# Analysis of Radioactivity Levels in Finished Cement in Chongqing

**DOI:** 10.3390/ma19194125

**Published:** 2026-09-27

**Authors:** Feng Wang, Tianya Huang, Shang Geng, Chuncen Wu, Guoqiang Wang

**Affiliations:** 1 Key Laboratory of Low-Grade Energy Utilization Technologies and Systems (Chongqing University), Ministry of Education, Chongqing 400044, China; 2 School of Energy and Power Engineering, Chongqing University, Chongqing 400044, China; 3 Chongqing Radiation Environment Supervision and Management Station, Chongqing 401120, China

**Keywords:** finished cement, radioactivity, gamma spectrometry, Pearson correlation coefficient

## Abstract

During the cement production process, certain materials with relatively high levels of natural radioactivity are commonly incorporated. Without control, this may lead to a residential environment with prolonged elevated radioactivity, posing a potential threat to human health. In this study, a systematic investigation of radioactivity levels was conducted on cement samples from the three major regions of Chongqing: the main urban area, northeastern Chongqing, and southeastern Chongqing. Key parameters, including the activity concentrations of the radionuclides ^226^Ra, ^232^Th, and ^40^K; external hazard index; internal hazard index; the radium equivalent activity; and the additional annual effective dose from external gamma radiation, were calculated. X-ray fluorescence (XRF) analysis was also performed to examine the chemical composition of typical samples. The research findings indicate that all 57 tested cement samples from 20 plants complied with the radiation safety requirements. Among the sampled products, the radioactivity levels followed the order: main urban area > northeastern region > southeastern region. Specifically, the mean radium equivalent activity of samples from the main urban area was approximately 13.85% and 31.43% higher than those from northeastern and southeastern Chongqing, respectively. Statistical tests indicated that this regional difference was mainly driven by ^226^Ra, while ^232^Th and ^40^K showed no significant regional differences. Through XRF analysis of cement products and mineral raw materials with varying radioactivity levels, combined with Pearson correlation analysis, the radium equivalent activity was found to be positively correlated with the contents of TiO_2_ and ZrO_2_. Further analysis of individual radionuclides suggested that this correlation was mainly driven by ^232^Th, with a secondary contribution from ^226^Ra, whereas ^40^K showed no significant correlation. These results provide supporting data for evaluating the natural radioactivity levels in cement products from Chongqing.

## 1. Introduction

In recent years, with growing public attention to climate change, low-carbon environments, healthy living, and housing conditions [1], cement production and usage have continued to rise. Each year, billions of tons of cement are introduced into daily life to meet the continuous development demands of infrastructure [2]. The variety of cement types has increased to meet modern construction requirements and advancements in construction technology. Many countries have developed a variety of special-purpose cements. These cements can be classified, based on the type and proportion of admixtures, into Portland cement, ordinary Portland cement, Portland slag cement, Portland pozzolana cement, Portland fly ash cement, and composite Portland cement. In China, the cement product structure is dominated by ordinary Portland cement, Portland slag cement, and composite Portland cement, with these three types accounting for 92–95% of the country’s cement output. Currently, different countries permit different types and quantities of admixtures in Portland cement series, and the nomenclature for cement types is not standardized. Consequently, the classification of Portland cement series varies among countries, although the understanding of their fundamental properties and application ranges remains consistent. Table 1 summarizes the characteristics and applications of some common cements in China.

With the advancement of global energy-saving and environmental protection concepts, people are increasingly concerned about the conservation of resources and the protection of the environment. In this context, cement—the essential binder of concrete, which is the world’s second most consumed material after water [3]—has seen widespread use of various industrial wastes in the production of building materials. In addition to the well-known hazards of chemicals such as formaldehyde, ammonia, and benzene, the natural radioactivity contained in some waste materials should not be overlooked. Radioactivity exists in nature, and like all building materials, cement also has radioactivity. Natural radioactivity refers to the instability of the atomic nuclei of certain elements in nature, which can spontaneously decay, meaning that the nucleus of one nuclide transforms into the nucleus of another nuclide, accompanied by the emission of radiation. The main components of building materials such as cement include ores, slag, and other solid wastes, many of which contain radioactive nuclides, making them potential sources of radiation in indoor environments [4].

Zhang [5] investigated radioactivity levels in building materials from Liaoning Province, China, finding that cement radioactivity followed the order Portland fly ash cement > composite Portland cement > Portland slag cement > ordinary Portland cement, with southern Chinese cements exhibiting higher radionuclide activity than northern ones. Chen [6] conducted systematic measurements on nine cement samples, analyzing ^226^Ra, ^232^Th, and ^40^K activity concentrations. The study revealed that ^40^K shows random distribution patterns, while Portland slag cement contains significantly higher levels of ^226^Ra and ^232^Th. The radioactivity ranking was established as: white Portland cement < composite Portland cement < ordinary Portland cement < Portland slag cement. Tang et al. [7] measured radionuclide concentrations in various cement products, demonstrating that external hazard index and internal hazard index follow a distinct trend: slag cement > fly ash cement > ordinary cement > composite cement > white cement. Notably, slag and fly ash cements exhibited substantially higher concentrations of ^226^Ra, ^232^Th, and ^40^K compared to other cement types.

Bajoga A.D. et al. [8] assessed natural radioactivity in raw materials (limestone, gypsum, fly ash, iron ore) from Nigeria’s Ashaka Cement Company. Using gamma spectrometry, the study found mean activity concentrations of ^238^U, ^232^Th, and ^40^K all below global averages. Correlation analysis revealed a strong relationship (0.95) between ^238^U and ^232^Th activities, with weak correlations for thorium–potassium and uranium–potassium pairs. Alonso M.M. et al. [9] systematically measured the natural radionuclide activity concentrations and chemical compositions of various cement-based materials, including 16 types of cement, 27 types of supplementary cementitious materials (SCMs), seven pigments, and six aggregates. The results indicate white cement has lower activity than conventional CEM I, while calcium aluminate cement shows elevated ^232^Th series activity. Among SCMs, fly ash dominates in U/Th series activity, and olive biomass ash leads in ^40^K content. Kadum A. et al. [10] used a NaI spectrometer to assess natural radioactivity in Algerian cements. Comparative analysis with UNSCEAR data confirmed below-average activity concentrations for ^226^Ra, ^232^Th, and ^40^K, with variations attributed to the geological diversity across sampling locations. Buranurak S. et al. [11] comprehensively evaluated radiological hazards in 25 Thai Portland cement samples. Their multi-parameter assessment (including dose rates, hazard indices, and cancer risks) confirmed compliance with international safety standards, with only one brand showing elevated radioactivity hypothesized to be due to rare earth minerals.

As a traditional heavy industrial base in southwestern China, Chongqing hosts large-scale coal-fired power, metallurgical, and phosphorus chemical industries. When local cement enterprises co-process bulk solid wastes, the fly ash, blast furnace slag, phosphogypsum, and other materials they consume are characterized by a complex waste composition and a regional geochemical background typical of the southwestern heavy industry. Previous studies have mainly focused on cement radioactivity in other regions, while systematic data for finished cement products in Chongqing remain limited. Therefore, based on the local industrial background of Chongqing, this study conducted extensive sampling, radiological measurements, and elemental analyses of cement products from multiple enterprises in Chongqing, aiming to assess the radioactivity levels of cement in the Chongqing region.

## 2. Sampling and Measurement

### 2.1. Sample Collection and Preparation Method

#### 2.1.1. Origin Selection

Based on the production scale, nature, and operational status of the enterprises, 20 cement manufacturers in Chongqing were selected. These enterprises are categorized into three main regions: the Chongqing main urban area, northeastern Chongqing, and southeastern Chongqing, referred to as Regions A, B, and C respectively. Each sample is coded according to the plant number and cement type produced. For example, “A1-1” indicates Sample 1 from Cement Plant 1 in the Chongqing main urban area. The geographical distribution of the selected enterprises is shown in Figure 1.

#### 2.1.2. Sample Preparation

Since the samples were all finished cement, we ensured that the finished cement from the 20 companies had similar production dates, trying to guarantee that the collected samples were produced at the same time. The collected samples were dried in an oven at 100 ± 5 °C for 2 h, sieved through a 100-mesh screen, and then cooled to room temperature in a desiccator. The treated cement samples were then packed tightly into standard sample containers, weighed using an electronic balance, and sealed for 21 days to allow the internal radionuclides to reach decay equilibrium before measurement.

### 2.2. Measurement Methods

The activity concentration of radionuclides was measured using an ultra-low background high-purity germanium γ-spectrometer (CANBERRA-BE5030P, Mirion Technologies (Canberra), Inc., Meriden, CT, USA). The detector uses the ultra-low background high-purity germanium γ-spectrometer, and the measurement time is set to 24 h. When no sample is placed, the empty box of the same specification is measured, and the local count rate of the experimental environment is recorded for background deduction. Based on the physical assumption of secular radioactive equilibrium within the uranium and thorium decay chains, this study utilizes their characteristic short-lived decay progeny for indirect quantification, thereby ensuring high resolution and low error in spectral analysis. Specifically, ^226^Ra was determined indirectly through its progeny ^214^Pb (351.9 keV) and ^214^Bi (609.3 keV), and the final activity concentration was taken as the average of the two line results. ^232^Th was determined indirectly through the 911.2 keV line of ^228^Ac, which is a well-resolved and widely used line for ^232^Th series measurement in building materials. ^40^K was measured directly through its 1460.8 keV line. Possible spectral interferences were minimized by energy calibration with a standard multi-nuclide source, periodic background subtraction, and manual verification of peak boundaries.

Because the cement samples have complex and variable matrices, it was difficult to find a certified solid standard source. Therefore, a source-free efficiency calibration method was adopted. The core of this method is to establish a geometric model that accurately reproduces the actual measurement conditions using the Geometry Composer software. As shown in Figure 2, the model defines the key parameters of the detection system, including the dimensions and position of the HPGe crystal, the materials and thicknesses of the detector end window and absorber layers, the inner and outer diameters and wall thickness of the standard sample container, and the actual filling height and density of the sample. These geometric and material parameters were then used to generate an efficiency–energy curve covering the full energy range for the specific sample, as shown in Figure 3. From this curve, the detection efficiency corresponding to any characteristic gamma-ray energy can be accurately obtained, thereby improving the accuracy and reliability of the activity calculation. According to GB 6566-2010 [12], when the sum of the activity concentrations of ^226^Ra ^232^Th, and ^40^K in a sample exceeds 37 Bq kg^−1^, the measurement uncertainty (coverage factor k = 1) should be ≤20%. In the present data evaluation, considering counting statistical fluctuations and detection efficiency errors, the relative expanded uncertainty (k = 2) of the target radionuclide activity concentrations in the samples did not exceed 20%, corresponding to a standard uncertainty (k = 1) of ≤10%, which meets the relevant requirements.

Activity Concentration: Based on the peak area, γ-ray detection efficiency, sample mass, counting time, and γ-ray emission probability, the activity concentrations of ^226^Ra, ^232^Th, and ^40^K were calculated using the following formula:

(1) $$C\;=\;\frac{N}{\varepsilon \mathrm{mt}\eta }$$

where C is represents activity concentration (Bq kg^−1^); N is the count of characteristic gamma rays; and ε is the detection efficiency of the characteristic gamma rays. Also m, t and η are sample mass (kg), measurement time (s) and gamma-ray emission probability.

External and internal hazard index: The degree of radiation hazard inside and outside the body is measured by the external and internal hazard indices, which must be less than 1 [12] according to the Limits of Radionuclides in Building Materials (GB 6566-2010). The formula for the internal hazard index is as follows:

(2) $$I_{\mathrm{in}}\;=\;\frac{C_{\mathrm{Ra}}}{200}$$

where Iin is the internal hazard index and CRa is the activity concentration of ^226^Ra in Bq kg^−1^. The formula for the external hazard index is as follows:

(3) $$I_{\mathrm{ex}\;}=\;\frac{C_{\mathrm{Ra}}}{370}+\frac{C_{\mathrm{Th}}}{260}+\frac{C_{K}}{4200}$$

where I_ex_ is the external hazard index. Also, CRa, CTh and CK are the activity concentrations of ^226^Ra, ^232^Th and ^40^K in Bq kg^−1^, respectively.

Radium Equivalent Activity Concentration: The distribution of ^226^Ra, ^232^Th, and ^40^K in various building materials varies significantly. To compare the relative γ radioactivity of different types of building materials, the radium equivalent activity concentration can be calculated. It is internationally recognized that 1 Bq kg^−1^ of ^226^Ra, 0.7 Bq kg^−1^ of ^232^Th, and 13 Bq kg^−1^ of ^40^K produce the same gamma dose rate [13]. Therefore, this study adopts the internationally used Beretka calculation model [14]:

(4) $${\mathrm{Ra}}_{\mathrm{eq}}\;={\;C}_{\mathrm{Ra}}\;+\;1.43C_{\mathrm{Th}}\;+\;0.077C_{K}$$

where Raeq is the radium equivalent activity concentration in Bq kg^−1^. Also, CRa, CTh and CK are the activity concentrations of ^226^Ra, ^232^Th and ^40^K in Bq kg^−1^, respectively. A commonly used reference level for radium equivalent activity in building materials is 370 Bq kg^−1^. This value is widely adopted as a screening level for comparing the relative gamma radioactivity of different building materials.

Additional annual effective dose from external gamma radiation: The additional annual effective dose from external gamma radiation was estimated using the room model proposed by the European Commission (RP112). The room dimensions are 4 m × 5 m × 2.8 m, with a wall thickness of 20 cm and a density of 2350 kg m^−3^ The formula for the absorbed dose rate of the entire room is shown below [15]:

(5) $$D\;=\;0.92C_{\mathrm{Ra}}\;+\;1.1C_{\mathrm{Th}}\;+\;0.08C_{K}$$

where D is the absorbed dose rate in nGy h^−1^. Also, C_Ra_, C_Th_ and C_K_ are the activity concentrations of ^226^Ra, ^232^Th and ^40^K in Bq kg^−1^. Buildings are the primary locations for human daily activities. Therefore, the indoor occupancy time is taken as 8760 h a^−1^, the conversion factor from absorbed dose in air to effective dose is 0.7 Sv Gy^−1^, and the indoor occupancy factor is 0.8. The formula for calculating the additional annual effective dose is as follows [16]:

(6) $$D_{E}\;=\;D\;\times \;0.7\;\times \;0.8\;\times \;8760\;\times \;10^{-6}\;$$

where D_E_ is the additional annual effective dose from external gamma radiation in the standard room model (mSv). This quantity represents the external gamma dose associated with the building material in the specified room scenario and does not include internal exposure pathways such as radon inhalation. According to the Basic standards for protection against ionizing radiation and for the safety of radiation sources, the annual effective dose limit for the public is 1 mSv [17]. This value is used here as a reference for the additional external gamma dose from building materials; it is not a direct regulatory limit for cement.

## 3. Results

### 3.1. Measurement Results

The results of the specific activity measurements are presented in Appendix A. The data are visualized as a box plot in Figure 4. While radioactivity levels varied among the samples, the overall trend consistently showed that C^40^K > C^226^Ra > C^232^Th. This distribution pattern can be attributed to the geochemical properties and elemental occurrence states of the natural raw materials used in cement production, which can be explained by three factors: the high abundance of potassium in clay minerals, the geochemical association between Ra and Ca, and the low mobility of Th in surface environments.

The high activity concentration of ^40^K originates from the high abundance of its parent element potassium in the Earth’s crust and its enrichment in clay-based raw materials. Potassium is one of the primary rock-forming elements in the crust and is widely present in auxiliary materials used in cement production, such as clay, shale, and fly ash. These materials are rich in potassium-bearing mineral phases like potassium feldspar, illite, and mica. Although the abundance of the radioactive isotope ^40^K in natural potassium is only 0.0117%, the immense total potassium content makes its overall activity contribution the most significant among the three radionuclides.

The activity concentration of ^226^Ra is intermediate, primarily due to its geochemical similarity to Ca. ^226^Ra is a daughter nuclide in the ^238^U decay series, and its activity depends on the content of its parent uranium in the raw materials. The key factor is that Ra and Ca both belong to the alkaline earth metal group and share similar chemical properties. This characteristic allows Ra^2+^ to be incorporated into limestone, the most important raw material in cement production, mainly through co-precipitation and adsorption onto calcium carbonate during rock formation. Consequently, this leads to the effective enrichment of ^226^Ra in the final cement product.

The lowest activity concentration of ^232^Th is associated with its low mobility in surface environments and the lack of effective enrichment mechanisms. Thorium ions exhibit strong geochemical inertness and weak migration capacity, typically being stably immobilized within the crystal lattices of weathering-resistant heavy minerals such as monazite and zircon. In cement raw materials, thorium is primarily introduced along with aluminosilicate components like clay. However, unlike potassium, it is not a major constituent, nor does it possess an enrichment pathway through isomorphic substitution akin to radium–calcium. Therefore, its activity concentration in the final product is generally the lowest among the three.

Based on the activity concentrations of radium, thorium, and potassium provided in Appendix A, parameters such as the external hazard index, internal hazard index, radium equivalent activity concentration, absorbed dose rate, and additional annual effective dose from external gamma radiation were calculated. The results are illustrated in Figure 5, with detailed data available in Appendix B. The calculated values are derived from the sample analysis data obtained during the project, with corresponding background contributions already subtracted during the analytical process.

Based on a systematic analysis of the radioactivity levels in cement samples from the main urban area, northeastern Chongqing, and southeastern Chongqing, variations were observed among different types of cement within the same enterprise. This is primarily due to the varying blending proportions of high-radioactivity raw materials in different cement types. Differences also exist among identical cement types from different enterprises, which is mainly attributed to the diverse geographical origins of their raw materials (such as limestone and industrial waste) and their correspondingly distinct natural radioactive backgrounds.

As shown in Figure 5, the radioactivity levels among enterprises in the northeastern and southeastern regions of Chongqing showed relatively minor variations, indicating a high degree of homogeneity. This may be attributed to similar raw material sources or more consistent geological backgrounds in these regions.

Despite the relative differences observed between regions and enterprises, all the tested samples exhibited radioactivity indices well below the strict national limits for building materials. This indicates that the overall radioactivity risk of cement products in Chongqing is relatively low, and the potential health impacts from additional radiation doses caused by these products are minimal.

### 3.2. Regional Comparative Analysis of Radioactive Levels

Figure 6 presents a regional comparison of box plots for the radiological assessment indices of cement samples from the Chongqing main urban area, northeastern Chongqing, and southeastern Chongqing. The figure clearly reveals a significant geographical spatial distribution pattern in the radioactivity levels of cement products in Chongqing. A stepwise gradient of “main urban area > northeastern region > southeastern region” is consistently observed across all indices, including the external hazard index, internal hazard index and additional annual effective dose from external gamma radiation.

The mean radium equivalent activity of the main urban area samples was higher than those of the other two regions. The mean radium equivalent activity reached 134.295 Bq kg^−1^; compared with the northeastern Chongqing region (mean of 117.963 Bq kg^−1^) and the southeastern Chongqing region (mean of 102.180 Bq kg^−1^), it was approximately 13.85% and 31.43% higher, respectively. At the same time, judging from the distribution shape of the box plots, the interquartile range and the range of samples in the main urban area are the largest, indicating that the radioactive levels of cement products in this region are not only overall higher but also significantly variable internally, with a more dispersed data distribution. In contrast, the radioactive levels of samples from northeastern and southeastern Chongqing are relatively concentrated and stable. The external hazard index, internal hazard index and the additional annual effective dose from external gamma radiation show a similar pattern to the radium equivalent activity.

To quantitatively examine the geographical differences observed in Figure 6, hypothesis testing was performed to determine whether the activity concentrations of ^226^Ra, ^232^Th, and ^40^K differed significantly among the main urban area, northeastern Chongqing, and southeastern Chongqing. Normality was first checked using the Shapiro–Wilk test. For ^226^Ra and ^40^K, the normality assumption was violated in at least one region, so the Kruskal–Wallis H test with Conover’s post hoc test was applied. For ^232^Th, both normality and homogeneity of variance (Levene’s test, *p* = 0.655) were satisfied, so ANOVA was used. The results showed significant regional differences for ^226^Ra (H = 15.01, *p* = 0.00055). Conover’s post hoc test indicated that both the main urban area and northeastern region were significantly higher than the southeastern region (*p* < 0.001 and *p* = 0.038, respectively), whereas the difference between the main urban area and northeastern region was not significant (*p* = 0.062). For ^232^Th (F(2,54) = 0.79, *p* = 0.458) and ^40^K (H = 0.28, *p* = 0.870), no statistically significant regional differences were found. Therefore, the observed regional gradient was mainly driven by ^226^Ra, while ^232^Th and ^40^K were relatively uniform among the sampled regions.

To visualize the distribution of the measured samples, GIS technology was used. The mean additional annual effective dose values of the cement samples from each district and county were classified and color-coded, as shown in Figure 7. Gray areas indicate districts where no cement samples were collected. Among the measured samples, relatively higher values were mainly observed in the main urban area, whereas lower values were generally found in southeastern Chongqing. The diagram is based only on the samples collected in this study and is intended as a schematic representation.

As the economic and industrial center, cement production in the core main urban area may rely more on raw materials sourced from specific mineral deposits or incorporate certain industrial by-products (such as fly ash, phosphogypsum, etc.), which may inherently possess a relatively higher natural radionuclide background. Conversely, in mountainous areas such as southeastern Chongqing, the demand for the co-disposal of industrial wastes is relatively low. Cement production in these regions relies primarily on natural mineral raw materials, and the geological structure of their source areas inherently dictates a lower natural radioactivity background. Regional differences may be associated with different origins of raw materials, geological background, and the use of industrial by-products, but their relative contribution cannot be determined from the existing data. 

All color grades on the map represent safety levels significantly below the relevant standard limits (1 mSv). The value of this map lies not in signaling risk, but in scientifically revealing the natural spatial distribution pattern of radioactivity levels in building materials. It provides a valuable data foundation and clear guidance for relevant departments to implement more targeted source quality control, for enterprises to optimize raw material procurement strategies, and for subsequent in-depth research.

### 3.3. Analysis of the Correlation Between Cement Elements and Radioactivity

To investigate the material basis for the differences in radioactivity levels among different cement samples, this study selected three types of cement: ordinary Portland cement (P.O), composite Portland cement (P.C), and masonry cement (M32.5). From the 57 cement samples collected in this study, nine typical samples representing high, medium, and low radioactivity levels were selected based on their radium equivalent activity. Their main chemical components were precisely measured using X-ray fluorescence spectroscopy (XRF), and the results are shown in Table 2. The aim is to analyze the intrinsic relationship between chemical composition and radioactivity levels.

Natural radionuclides ^226^Ra and ^232^Th are present in extremely low concentrations in nature and cannot be directly detected by conventional XRF. However, their geochemical behavior often associates them with certain trace or minor elements. Therefore, by analyzing the variations in the concentrations of these carrier or indicator elements, the enrichment of ^226^Ra and ^232^Th can be inferred indirectly.

By comparing the chemical compositions of samples with high, medium, and low radioactivity levels, the study found that titanium (TiO_2_) and zirconium (ZrO_2_) are strongly correlated indicators of high radioactivity. The most significant pattern identified is that all samples with the highest radioactivity levels (A12 series) exhibited substantially higher contents of titanium dioxide (TiO_2)_ and zirconium dioxide (ZrO_2_) compared to samples with the lowest radioactivity levels. For instance, in ordinary Portland cement, the highly radioactive sample A12-5 contained 0.536% TiO_2_ and 0.0258% ZrO_2_, whereas the low radioactivity sample C2-2 contained only 0.283% and 0.0105%, respectively. This pattern also held true in comparisons between composite Portland cement and masonry cement.

In nature, Ti- and Zr-bearing accessory minerals are common in rocks, such as rutile/ilmenite and zircon. These accessory minerals may incorporate U and Th ions because of similar crystallochemical properties, and they may therefore be associated with the enrichment of U and Th in the studied samples.

To further examine the above association, raw mineral materials with varying radioactivity levels from cement production were selected for XRF testing. The specific samples selected, along with their corresponding radium equivalent activity, TiO_2_, and ZrO_2_ contents, are presented in Table 3 [1]. The data from this table, combined with the finished cement data from Table 2 mentioned above, were used to generate the radioactivity level and element correlation analysis diagram shown in Figure 8.

As indicated in Figure 8, the contents of TiO_2_ and ZrO_2_ show a positive correlation with the radium equivalent activity. To validate the aforementioned conclusion, the correlations among the three parameters radium equivalent activity, TiO_2_ content, and ZrO_2_ content were analyzed. The Pearson correlation coefficient (r) and its significance test (*p*-value) were calculated for each pair, with the results presented in Table 4 below.

Generally, a *p* < 0.05 indicates a statistically significant correlation, with higher r-values denoting stronger correlations. The results of the Pearson correlation analysis demonstrate that the radium equivalent activity exhibits a strong, statistically significant positive correlation with both the TiO_2_ and ZrO_2_ contents. Furthermore, an approximate linear correlation exists between the two independent variables, TiO_2_ and ZrO_2_. Therefore, based on the samples in this study, the TiO_2_ and ZrO_2_ contents are presumed to show a strong correlation with the radioactivity levels.

As shown in Figure 8, the TiO_2_ and ZrO_2_ contents are positively correlated with the radium equivalent activity (Raeq). Because Raeq is a composite index of ^226^Ra, ^232^Th, and ^40^K, the correlations with individual radionuclides were further examined, as shown in Figure 9. ^232^Th shows strong positive correlations with TiO_2_ (r = 0.982, *p* = 5.29 × 10^−10^) and ZrO_2_ (r = 0.970, *p* = 9.33 × 10^−9^). ^226^Ra also shows significant positive correlations with TiO_2_ (r = 0.796, *p* = 6.53 × 10^−4^) and ZrO_2_ (r = 0.813, *p* = 4.13 × 10^−4^). In contrast, ^40^K shows no significant correlation with TiO_2_ or ZrO_2_. These results suggest that the correlation between Raeq and TiO_2_/ZrO_2_ is mainly driven by ^232^Th, with a secondary contribution from ^226^Ra.

## 4. Conclusions

Based on a systematic analysis of radiological levels in finished product samples from 20 cement enterprises across the three major regions of Chongqing—the main urban area, northeastern Chongqing, and southeastern Chongqing—key parameters including activity concentrations, external hazard index, internal hazard index, radium equivalent activity, and additional annual effective dose from external gamma radiation were calculated. Combined with the XRF chemical composition analysis of representative samples and analyzing only the detection data from the samples collected in this study, the following conclusions were drawn:

(1) Significant geographical spatial distribution characteristics exist in radioactivity levels. The main urban area generally showed higher radioactivity levels than northeastern and southeastern Chongqing, following the order main urban area > northeastern region > southeastern region. Statistical tests indicated that this regional difference was mainly driven by ^226^Ra, while ^232^Th and ^40^K showed no significant regional differences. This distribution may be associated with regional geological backgrounds and industrial layouts, where urban areas may incorporate more industrial wastes with slightly higher natural backgrounds compared to the natural minerals used in southeastern mountainous areas.
(2) A possible association between radioactivity levels and Ti/Zr contents was observed. Through XRF analysis of representative samples with high, medium, and low radioactivity levels, combined with Pearson correlation analysis, the radium equivalent activity was found to be positively correlated with TiO_2_ and ZrO_2_ contents. Further analysis of individual radionuclides indicated that this correlation was mainly associated with ^232^Th, with a secondary contribution from ^226^Ra, and was not significant for ^40^K. Therefore, based on the samples in this study, the TiO_2_ and ZrO_2_ contents are presumed to show a strong correlation with the radioactivity levels.
(3) All tested cement products demonstrated excellent safety performance. Despite the relative differences observed among regions and enterprises, relevant radioactivity indices of the cement samples from all tested enterprises across the three regions were significantly below the limit values specified for building materials in the Chinese national standard GB 6566-2010. Among them, cement samples from southeastern Chongqing exhibited the overall lowest radioactivity levels and the largest safety margin.

## Figures and Tables

**Figure 1:**
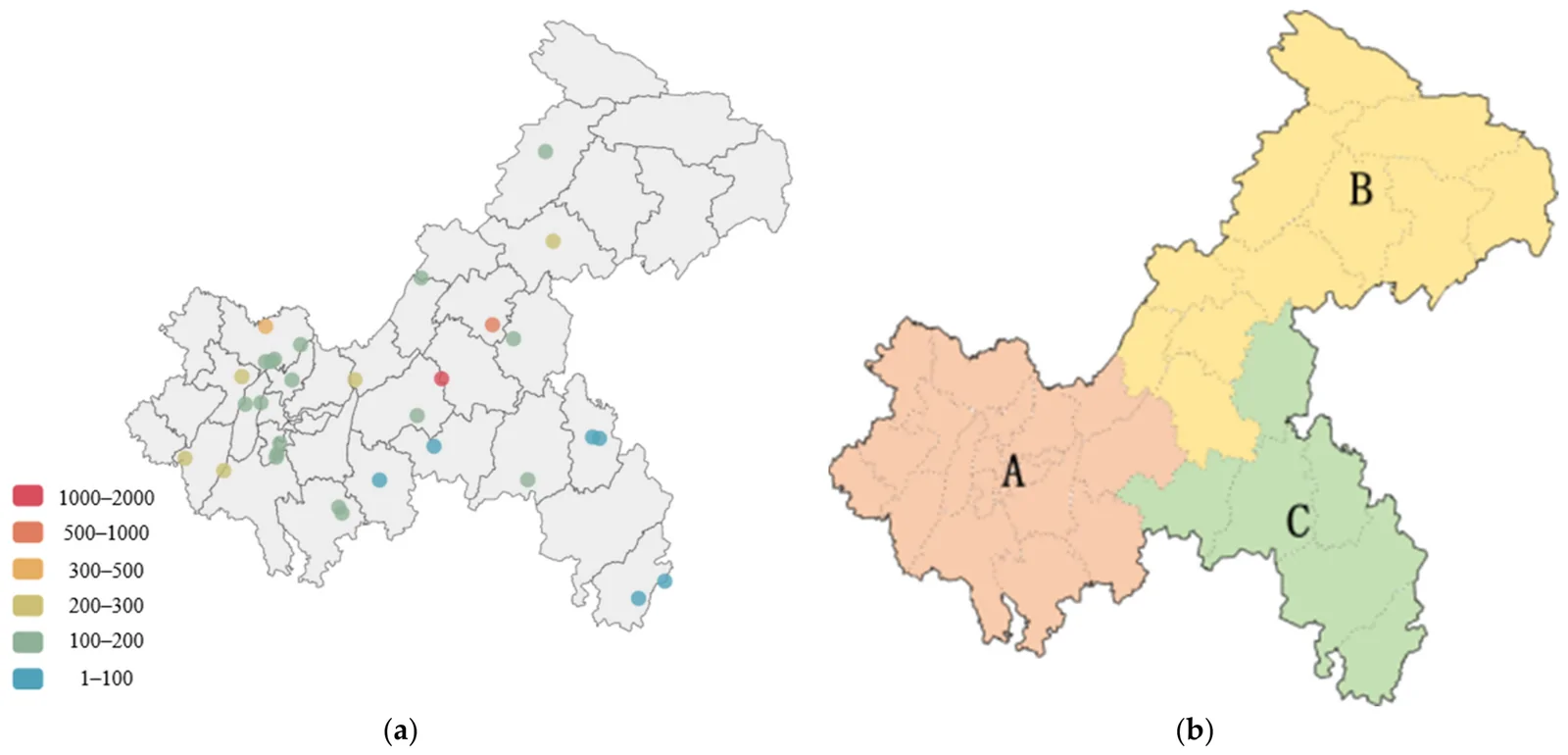
Chongqing cement industries: (**a**) cement production capacity (ten thousand tons) distribution map and (**b**) sampling area division map.

**Figure 2:**
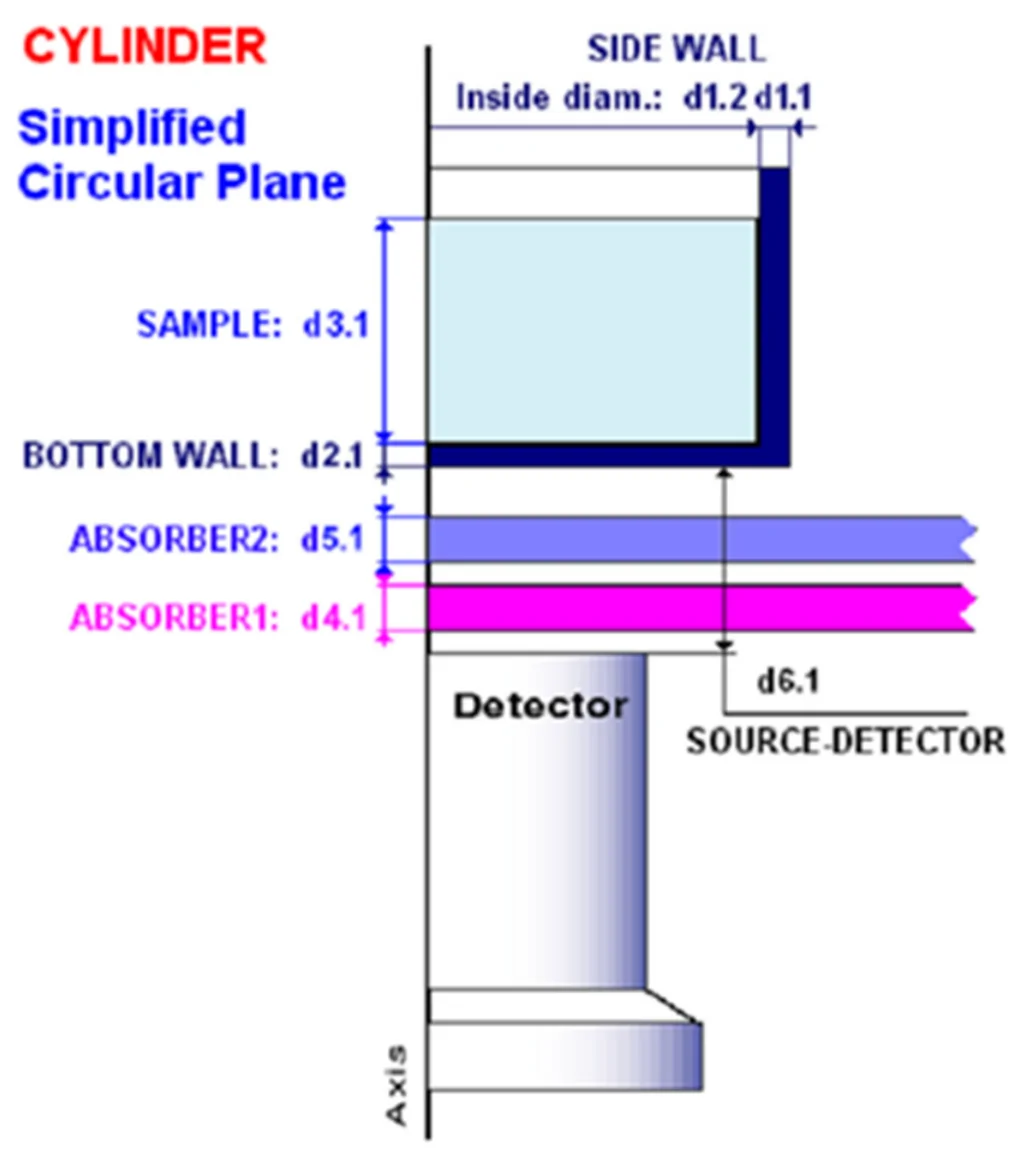
A schematic diagram of the geometric model for detection efficiency.

**Figure 3:**
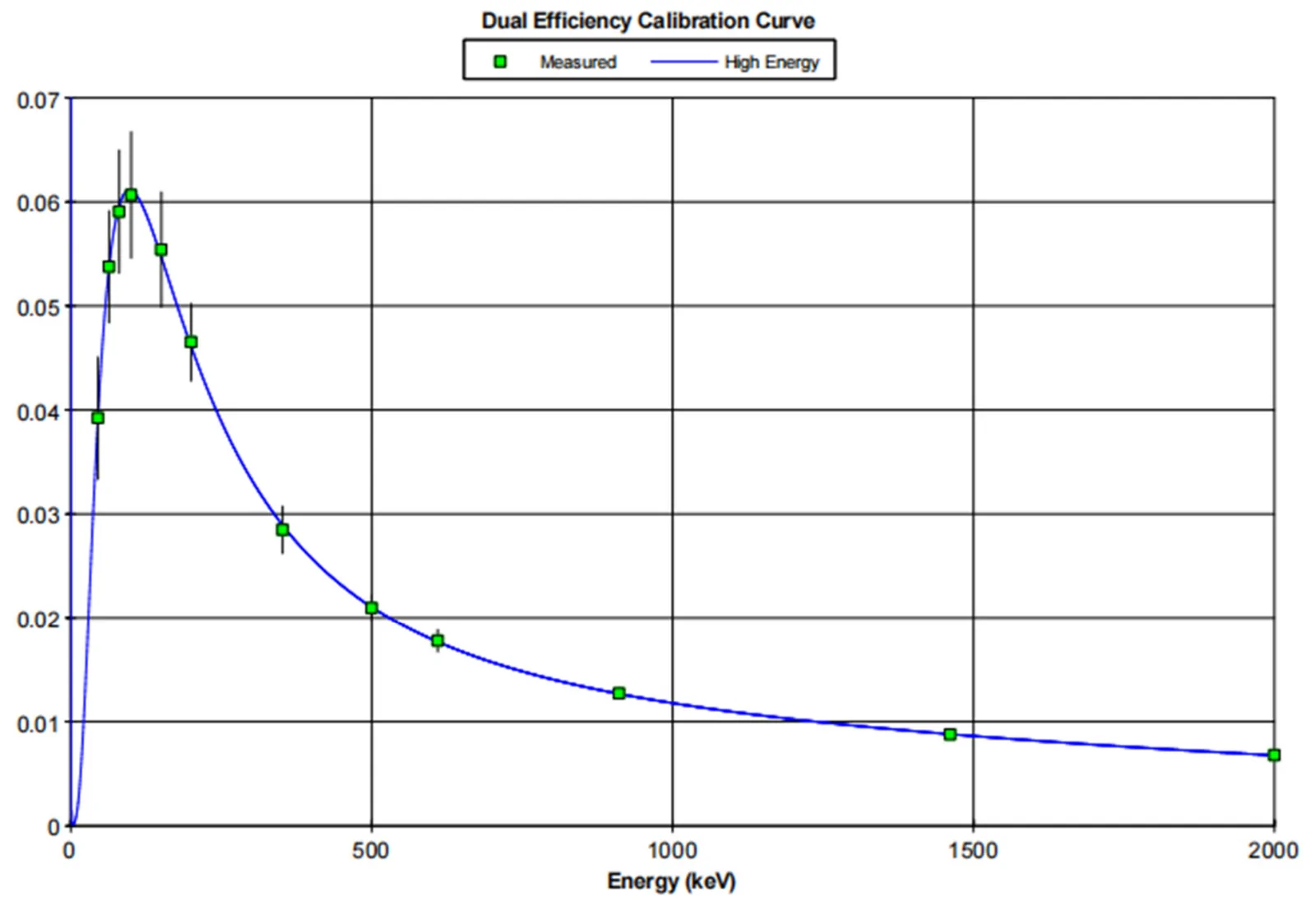
Detection efficiency–energy relationship curve.

**Figure 4:**
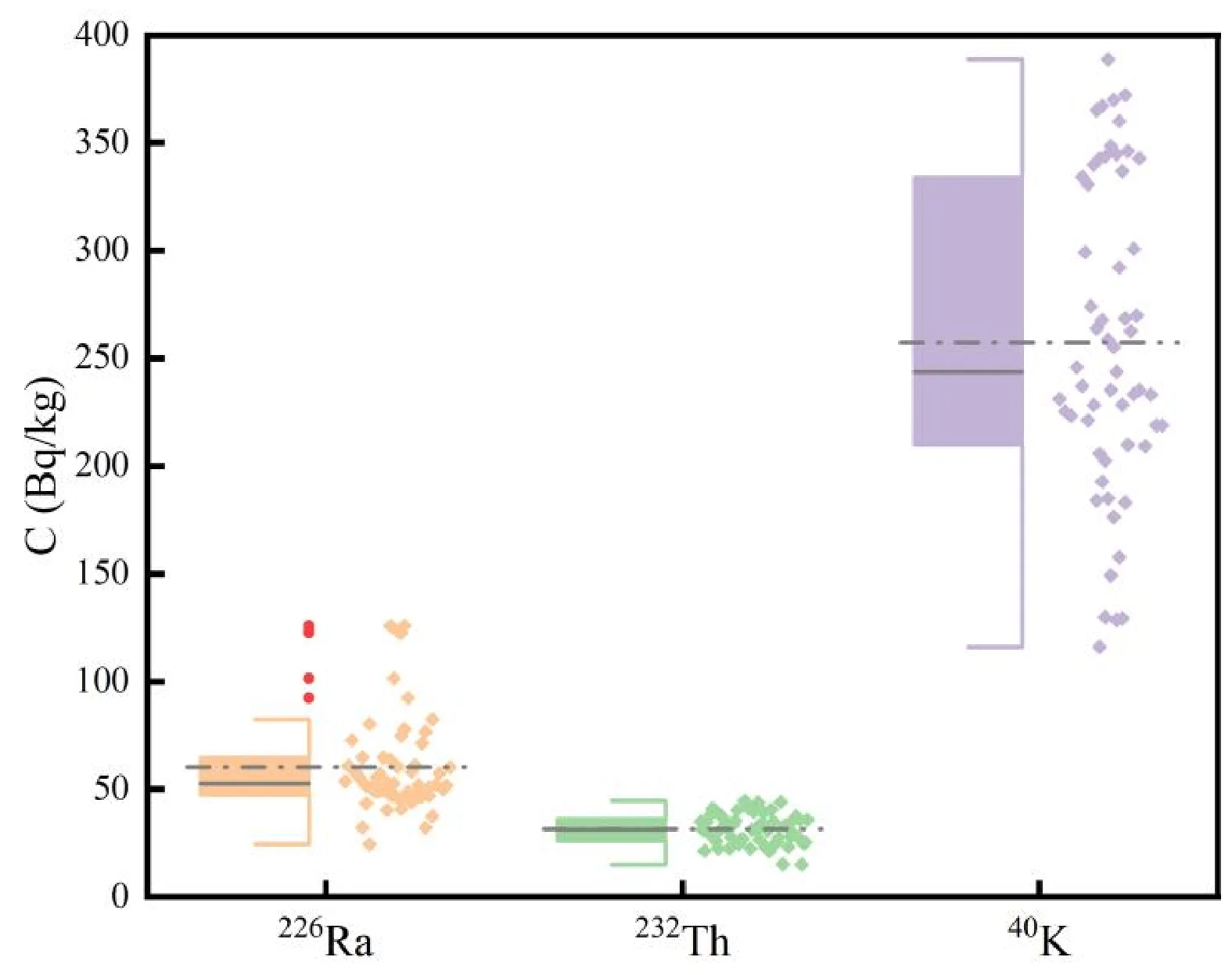
Box plot of nuclide specific activity.

**Figure 5:**
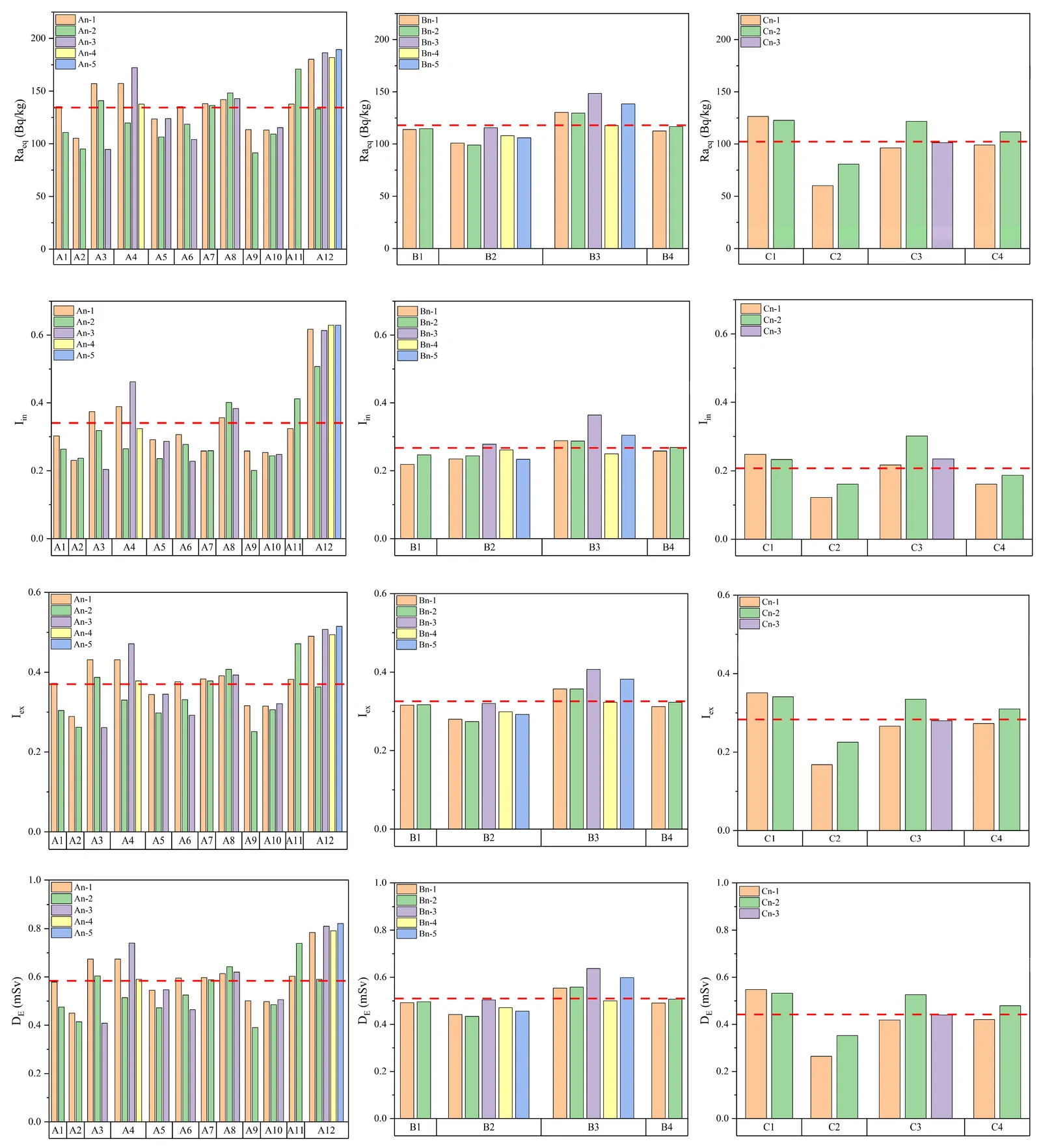
Data charts of radioactive evaluation indicators in the main urban area, northeastern Chongqing, and southeastern Chongqing.

**Figure 6:**
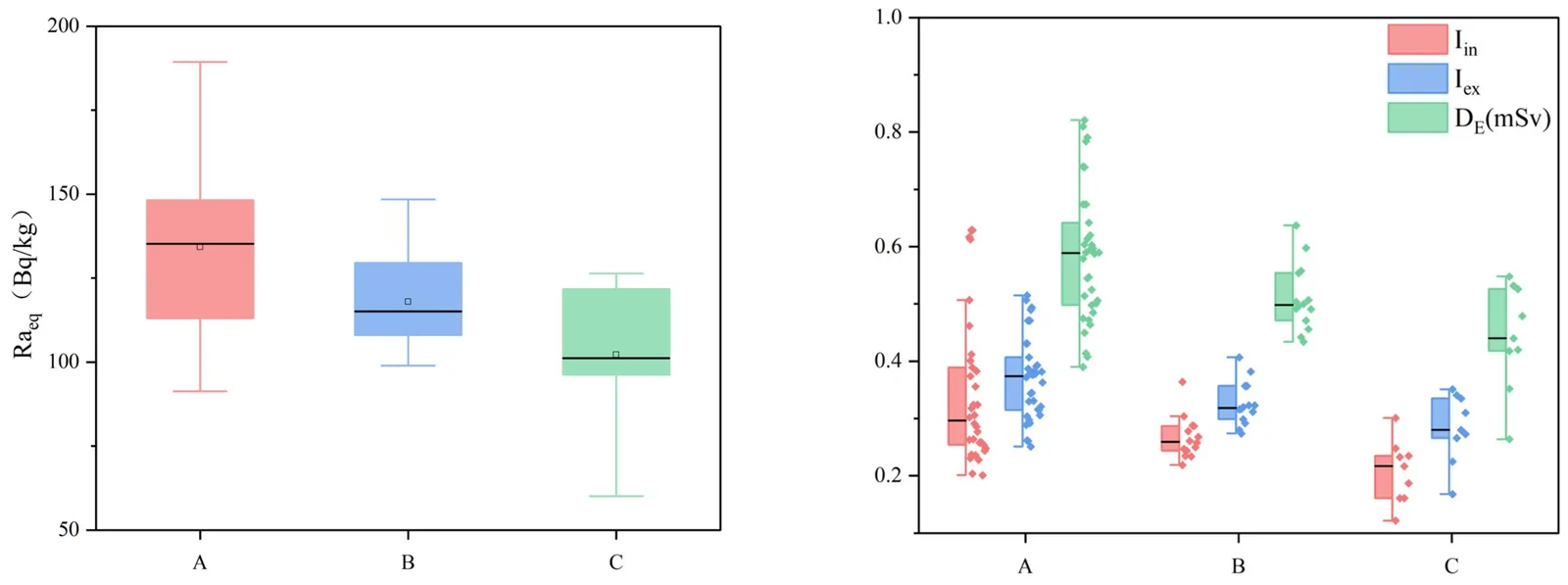
Comparison box plot of radioactive evaluation indices of samples in different regions.

**Figure 7:**
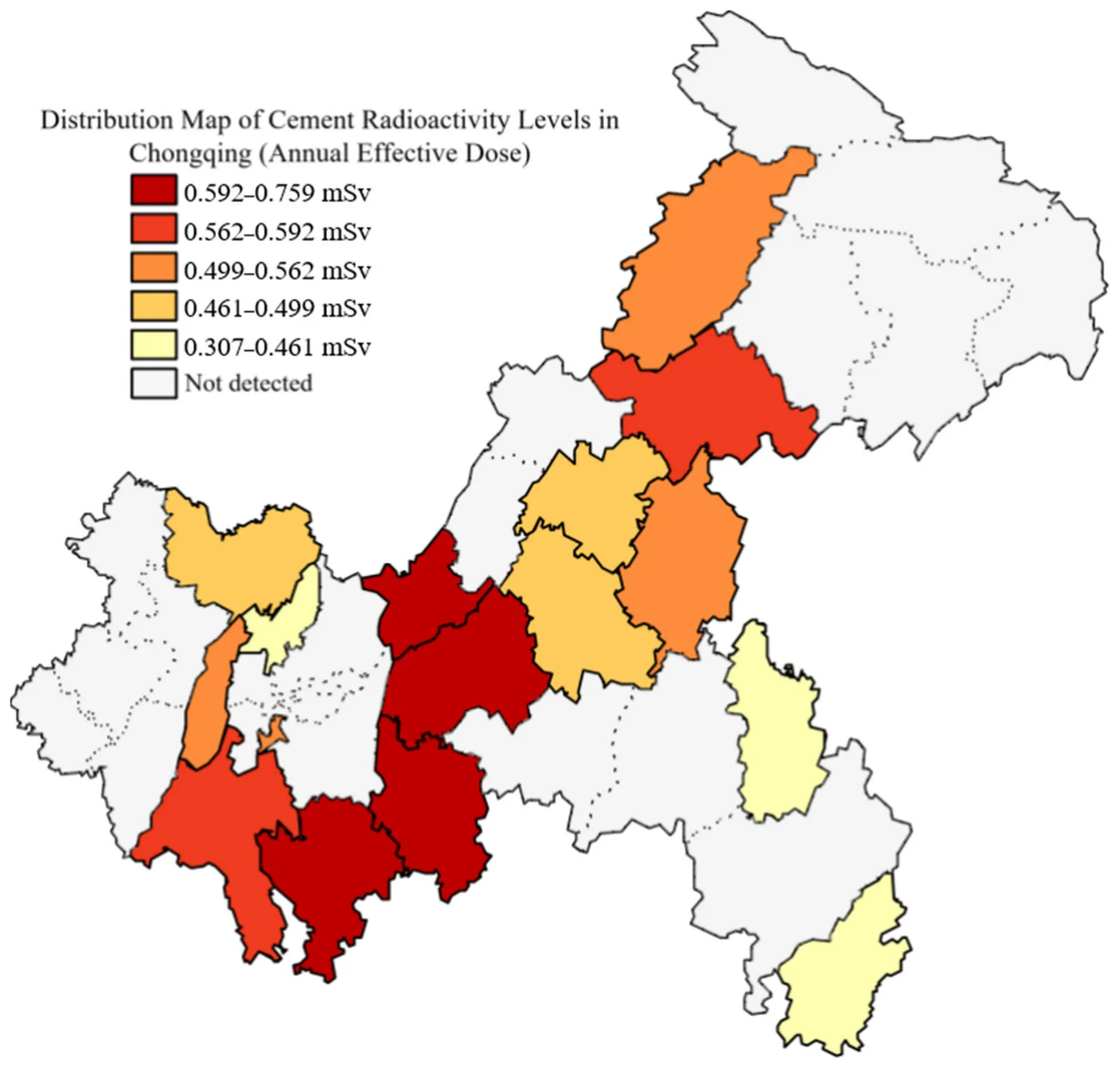
Radioactive distribution diagram of cement samples in Chongqing.

**Figure 8:**
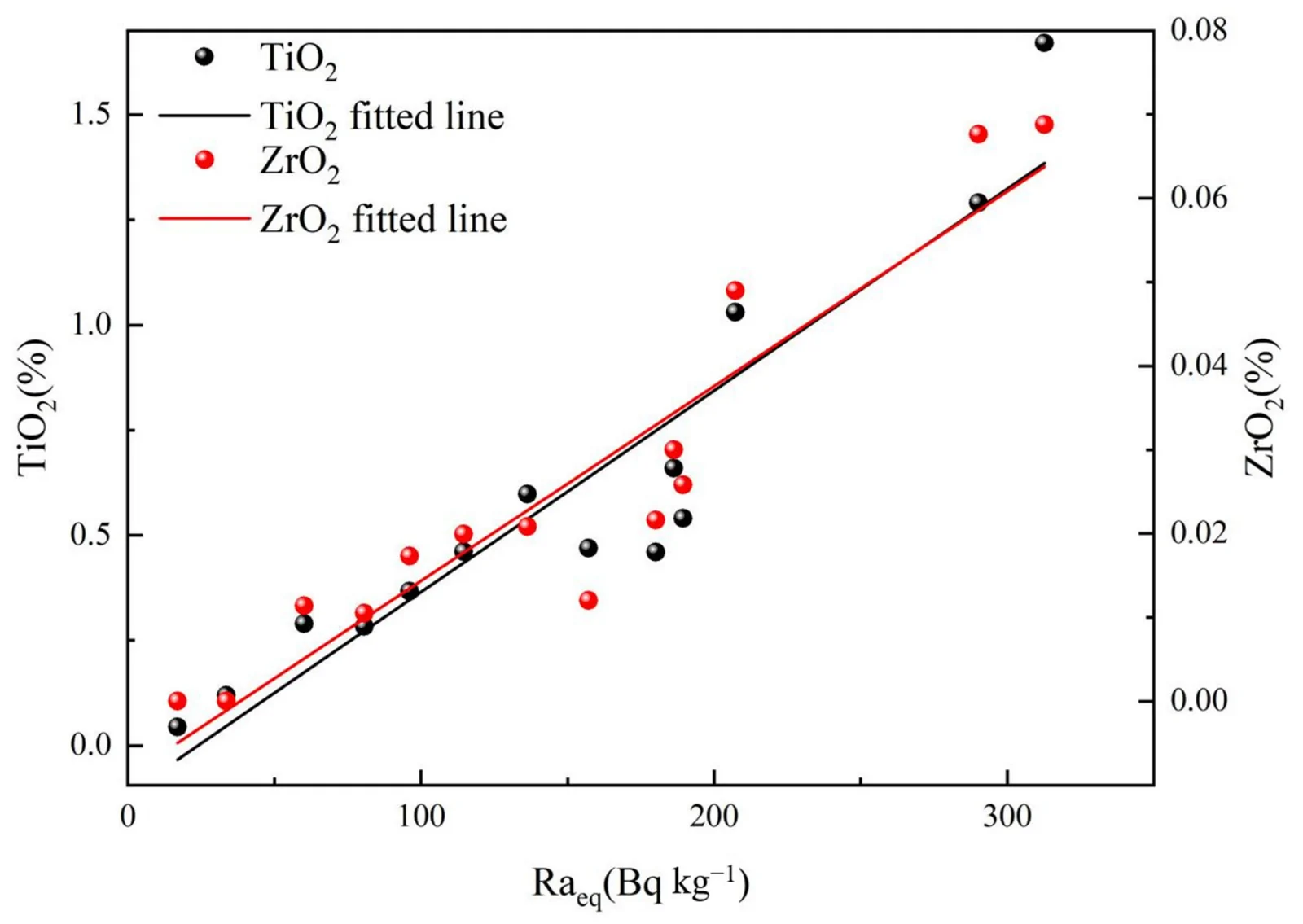
Correlation analysis of radium equivalent activity with TiO_2_ and ZrO_2_ content.

**Figure 9:**
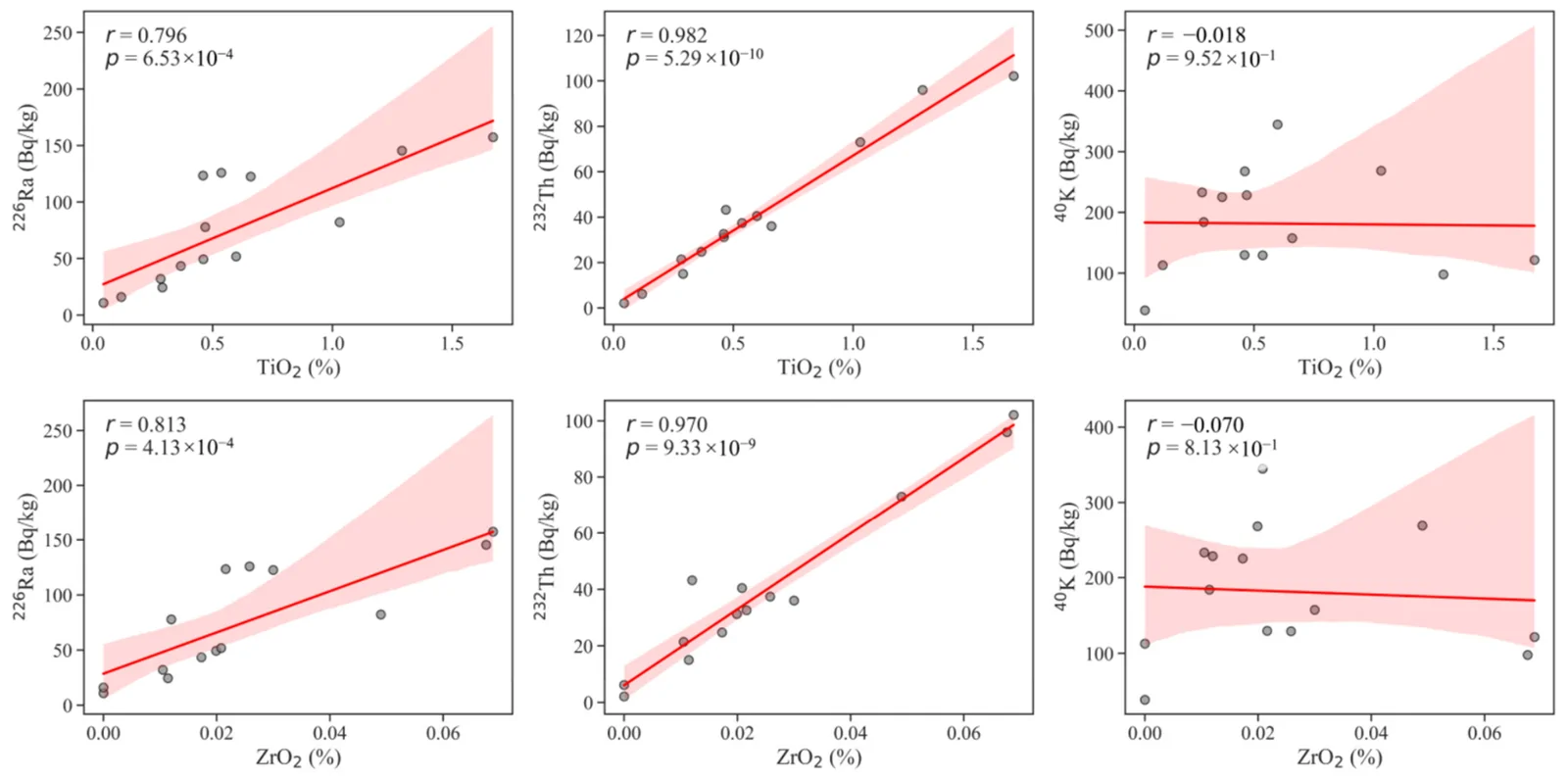
Correlation analysis between radionuclides and TiO_2_, ZrO_2_ content.

**Table 1:** Characteristics of common Portland cements.

Name	Composition	Characteristics	Code
Portland Cement	Portland cement clinker + 0–5% blending materials + appropriate amount of gypsum	High strength, rapid hardening, good frost/wear resistance. Disadvantages: High hydration heat, weak chemical resistance.	P.I P.II
Ordinary Portland Cement	Portland cement clinker + 5–20% blending materials + appropriate amount of gypsum	Excellent comprehensive performance and high early strength. Disadvantages: Relatively high hydration heat and poor chemical resistance.	P.O
Portland Slag Cement	Portland cement clinker + 20–70% granulated blast furnace slag + appropriate amount of gypsum	Low hydration heat, strong sulfate resistance, rapid late-strength development. Disadvantages: Low early strength, poor water retention, large drying shrinkage.	P.S
Composite Portland Cement	Portland cement clinker + 20–50% of ≥2 types of blending materials + appropriate amount of gypsum	Low hydration heat, dense structure, good workability. Disadvantages: Low early strength, poor frost/wear resistance (varies with blending materials).	P.C
Masonry Cement	Portland cement clinker + large amount of blending materials (>50%) + appropriate amount of gypsum	Excellent water retention, workability, and bonding; small shrinkage. Disadvantages: Low strength. Prohibited for load-bearing structures or freeze–thaw environments.	M

**Table 2:** Cement element content of different radioactive levels.

Types of Cement	Sample Number	Radium Equivalent Activity (Bq kg^−1^)	TiO_2_ (%)	ZrO_2_ (%)
Ordinary Portland Cement P.O	A12-5	189.390	0.536	0.0258
	A4-1	157.250	0.469	0.0120
	C2-2	80.695	0.283	0.0105
Composite Portland Cement P.C	A12-1	180.157	0.460	0.0216
	A7-2	136.289	0.598	0.0208
	C3-1	96.233	0.367	0.0173
Masonry Cement M	A12-3	186.249	0.659	0.0300
	B1-2	114.648	0.461	0.0199
	C2-1	60.119	0.290	0.0114

**Table 3:** Element content of raw materials for cement production with different radioactivity levels.

Sample Name	Ra_eq_ (Bq kg^−1^)	TiO_2_ (%)	ZrO_2_ (%)
Desulfurization Gypsum	16.925	0.0442	Not detected
Limestone Raw Material	33.646	0.119	Not detected
Coal Slag	207.331	1.03	0.0490
Slag	290.227	1.29	0.0676
Blast Furnace Slag Composite Material	312.726	1.67	0.0688

**Table 4:** Pearson correlation coefficient r and its significance test *p* results.

Project	n	Pearson Correlation Coefficient r	Significance Test *p*
Ra_eq_vsTiO_2_	14	0.937	8.2141 × 10^−7^
Ra_eq_vsZrO_2_	14	0.936	8.3166 × 10^−7^
TiO_2_vsZrO_2_	14	0.979	1.3306 × 10^−9^

## Data Availability

The data presented in this study are available on reasonable request from the corresponding author. Further inquiries can be directed to the corresponding author.

## Appendix A

**Table A1:** Radioactive nuclide content in cement samples from Chongqing.

No.	Name	C^226^Ra (Bq kg^−1^)	C^232^Th (Bq kg^−1^)	C^40^K (Bq kg^−1^)
A1-1	P.O42.5R	60.377 ± 8.597	39.655 ± 3.659	235.201 ± 20.112
A1-2	P.L32.5R	52.638 ± 7.521	31.134 ± 3.066	176.399 ± 15.638
A2-1	M32.5	46.135 ± 6.548	31.373 ± 2.898	184.890 ± 15.699
A2-2	P.O42.5	47.344 ± 6.692	22.425 ± 2.200	202.344 ± 17.049
A3-1	P.O42.5R	74.826 ± 10.667	43.750 ± 4.109	255.268 ± 22.033
A3-2	P.C42.5	63.588 ± 9.059	40.950 ± 3.859	243.690 ± 21.070
A3-3	M32.5	40.893 ± 5.870	26.498 ± 2.799	205.763 ± 18.154
A4-1	P.O42.5	77.845 ± 11.099	43.226 ± 4.011	228.454 ± 19.788
A4-2	P.O52.5	52.898 ± 7.566	34.443 ± 3.383	228.336 ± 19.708
A4-3	P.C42.5	92.330 ± 13.134	44.607 ± 4.180	209.901 ± 18.397
A4-4	M32.5	64.705 ± 9.256	39.044 ± 3.725	221.179 ± 19.261
A5-1	P.C42.5R	58.189 ± 8.306	26.985 ± 2.758	348.345 ± 29.281
A5-2	M32.5	47.168 ± 6.767	22.970 ± 2.451	343.594 ± 28.989
A5-3	P.O42.5R	57.112 ± 8.148	26.759 ± 2.768	369.876 ± 31.001
A6-1	P.O42.5R	61.142 ± 8.747	30.843 ± 3.107	388.831 ± 32.613
A6-2	P.C42.5R	55.461 ± 7.939	24.727 ± 2.701	360.042 ± 30.309
A6-3	M32.5	45.573 ± 6.569	21.136 ± 2.412	367.162 ± 31.001
A7-1	P.O42.5	51.677 ± 7.397	40.326 ± 3.771	372.176 ± 31.150
A7-2	P.C42.5R	51.853 ± 7.412	40.480 ± 3.756	344.789 ± 28.961
A8-1	P.O42.5	71.254 ± 10.163	35.457 ± 3.487	258.663 ± 22.192
A8-2	M32.5	80.289 ± 11.433	34.926 ± 3.399	233.328 ± 20.104
A8-3	P.C42.5	76.603 ± 10.905	33.574 ± 3.244	237.145 ± 20.346
A9-1	9A-30	51.544 ± 7.362	24.842 ± 2.567	342.666 ± 28.741
A9-2	21A-14	40.259 ± 5.775	27.662 ± 2.746	149.157 ± 13.381
A10-1	P.C42.5R	50.703 ± 7.268	25.448 ± 2.672	336.827 ± 28.483
A10-2	P.O42.5R	48.771 ± 7.017	22.570 ± 2.435	365.126 ± 30.669
A10-3	M32.5	49.566 ± 7.088	27.667 ± 2.894	339.792 ± 28.690
A11-1	P.O42.5R	64.713 ± 9.257	32.419 ± 3.173	346.094 ± 29.178
A11-2	M32.5	82.421 ± 11.741	44.052 ± 4.092	330.603 ± 28.021
A12-1	P.C42.5	123.488 ± 17.527	32.635 ± 3.283	129.875 ± 12.130
A12-2	P.LH42.5	101.394 ± 14.396	15.203 ± 2.058	128.490 ± 11.948
A12-3	M32.5	122.596 ± 17.406	36.025 ± 3.560	157.625 ± 14.361
A12-4	P.O52.5R	125.869 ± 17.916	32.881 ± 3.674	115.961 ± 11.596
A12-5	P.O42.5R	125.876 ± 17.866	37.455 ± 3.615	129.254 ± 12.072
B1-1	P.O42.5	43.886 ± 6.278	33.231 ± 3.160	292.107 ± 24.778
B1-2	M32.5	49.358 ± 7.061	31.240 ± 3.061	267.751 ± 22.839
B2-1	P.II52.5R	46.945 ± 6.736	23.202 ± 2.415	268.532 ± 22.943
B2-2	M32.5	48.840 ± 7.017	22.391 ± 2.449	235.085 ± 20.380
B2-3	P.O42.5R	55.516 ± 7.939	27.801 ± 2.790	263.840 ± 22.506
B2-4	P.C42.5R	52.195 ± 7.458	25.793 ± 2.675	245.862 ± 21.130
B2-5	P.O42.5 (Iron Standard [18])	46.862 ± 6.702	30.045 ± 2.913	209.125 ± 18.146
B3-1	P.C42.5	57.597 ± 8.199	40.490 ± 3.798	192.810 ± 16.962
B3-2	M32.5	57.488 ± 8.211	36.256 ± 3.467	262.580 ± 22.398
B3-3	P.O42.5	72.757 ± 10.366	40.914 ± 3.817	223.267 ± 19.271
B3-4	P.O42.5 (Iron Standard)	50.071 ± 7.152	37.443 ± 3.489	183.069 ± 16.060
B3-5	P.II52.5	60.820 ± 8.695	38.067 ± 3.607	300.690 ± 25.506
B4-1	M32.5	51.585 ± 7.387	27.835 ± 2.794	274.062 ± 23.266
B4-2	P.O42.5	53.627 ± 7.639	29.564 ± 2.867	269.798 ± 22.904
C1-1	P.C42.5	49.599 ± 7.095	35.706 ± 3.407	334.092 ± 28.166
C1-2	P.O42.5	46.555 ± 6.672	34.758 ± 3.257	342.820 ± 28.734
C2-1	M32.5	24.471 ± 3.509	15.020 ± 1.693	184.029 ± 15.624
C2-2	P.O42.5	32.111 ± 4.573	21.429 ± 2.090	232.996 ± 19.381
C3-1	P.C42.5R	43.492 ± 6.175	24.748 ± 2.339	225.336 ± 18.849
C3-2	M32.5	60.239 ± 8.518	31.224 ± 2.876	218.889 ± 18.396
C3-3	P.O42.5R	47.044 ± 6.666	25.401 ± 2.444	231.077 ± 19.262
C4-1	P.O42.5	32.250 ± 4.592	34.884 ± 3.148	218.925 ± 18.321
C4-2	M32.5	37.445 ± 5.314	35.774 ± 3.241	299.199 ± 24.815

## Appendix B

**Table A2:** Radioactivity evaluation indicators for cement samples from Chongqing.

No.	Name	I_in_	I_ex_	Ra_eq_ (Bq kg^−1^)	D (nGy h^−1^)	D_E_ (mSv)
A1-1	P.O42.5R	0.302	0.372	135.194	117.984	0.579
A1-2	P.L32.5R	0.263	0.304	110.743	96.787	0.475
A2-1	M32.5	0.231	0.289	105.235	91.746	0.450
A2-2	P.O42.5	0.237	0.262	94.993	84.412	0.414
A3-1	P.O42.5R	0.374	0.431	157.044	137.387	0.674
A3-2	P.C42.5	0.318	0.387	140.910	123.040	0.604
A3-3	M32.5	0.204	0.261	94.630	83.231	0.408
A4-1	P.O42.5	0.389	0.431	157.250	137.443	0.674
A4-2	P.O52.5	0.264	0.330	119.734	104.821	0.514
A4-3	P.C42.5	0.462	0.471	172.281	150.804	0.740
A4-4	M32.5	0.324	0.378	137.569	120.171	0.590
A5-1	P.C42.5R	0.291	0.344	123.601	111.085	0.545
A5-2	M32.5	0.236	0.298	106.471	96.149	0.472
A5-3	P.O42.5R	0.286	0.345	123.858	111.568	0.547
A6-1	P.O42.5R	0.306	0.376	135.188	121.285	0.595
A6-2	P.C42.5R	0.277	0.331	118.544	107.027	0.525
A6-3	M32.5	0.228	0.292	104.068	94.549	0.464
A7-1	P.O42.5	0.258	0.383	138.000	121.675	0.597
A7-2	P.C42.5R	0.259	0.378	136.289	119.816	0.588
A8-1	P.O42.5	0.356	0.391	141.874	125.249	0.614
A8-2	M32.5	0.401	0.407	148.199	130.951	0.642
A8-3	P.C42.5	0.383	0.393	142.874	126.378	0.620
A9-1	9A-30	0.258	0.316	113.453	102.160	0.501
A9-2	21A-14	0.201	0.251	91.301	79.399	0.390
A10-1	P.C42.5R	0.254	0.315	113.030	101.586	0.498
A10-2	P.O42.5R	0.244	0.306	109.160	98.906	0.485
A10-3	M32.5	0.248	0.321	115.293	103.217	0.506
A11-1	P.O42.5R	0.324	0.382	137.721	122.884	0.603
A11-2	M32.5	0.412	0.471	170.871	150.732	0.739
A12-1	P.C42.5	0.617	0.490	180.157	159.898	0.784
A12-2	P.LH42.5	0.507	0.363	133.027	120.284	0.590
A12-3	M32.5	0.613	0.507	186.249	165.026	0.810
A12-4	P.O52.5R	0.629	0.494	181.817	161.245	0.791
A12-5	P.O42.5R	0.629	0.515	189.390	167.347	0.821
B1-1	P.O42.5	0.219	0.316	113.898	100.297	0.492
B1-2	M32.5	0.247	0.317	114.648	101.194	0.496
B2-1	P.II52.5R	0.235	0.280	100.801	90.194	0.442
B2-2	M32.5	0.244	0.274	98.960	88.369	0.434
B2-3	P.O42.5R	0.278	0.320	115.588	102.763	0.504
B2-4	P.C42.5R	0.261	0.299	108.010	96.060	0.471
B2-5	P.O42.5 (Iron Standard)	0.234	0.292	105.929	92.892	0.456
B3-1	P.C42.5	0.288	0.357	130.344	112.953	0.554
B3-2	M32.5	0.287	0.357	129.553	113.777	0.558
B3-3	P.O42.5	0.364	0.407	148.455	129.803	0.637
B3-4	P.O42.5 (Iron Standard)	0.250	0.323	117.711	101.899	0.500
B3-5	P.II52.5	0.304	0.382	138.409	121.884	0.598
B4-1	M32.5	0.258	0.312	112.492	100.002	0.491
B4-2	P.O42.5	0.268	0.323	116.678	103.441	0.507
C1-1	P.C42.5	0.248	0.351	126.384	111.636	0.548
C1-2	P.O42.5	0.233	0.341	122.657	108.491	0.532
C2-1	M32.5	0.122	0.168	60.119	53.757	0.264
C2-2	P.O42.5	0.161	0.225	80.695	71.754	0.352
C3-1	P.C42.5R	0.217	0.266	96.233	85.262	0.418
C3-2	M32.5	0.301	0.335	121.744	107.278	0.526
C3-3	P.O42.5R	0.235	0.280	101.161	89.708	0.440
C4-1	P.O42.5	0.161	0.273	98.991	85.556	0.420
C4-2	M32.5	0.187	0.310	111.639	97.736	0.479

## References

[1] Wang F., Geng S., Wu C.C., Du H.Y., Zhang X.Q., Song Y.F., Chen Y., Wang M.X., Wang G.Q., Huang T.Y. (2025). Analysis of cement raw materials radioactivity in Chongqing. Appl. Radiat. Isot..

[2] Zhang Z.Y., Fu S.J. (2022). A Brief Review of International Cement Industry Development and Carbon Emission Reduction Status. China Cem..

[3] Scrivener K.L., John V.M., Gartner E.M. (2018). Eco-efficient cements: Potential economically viable solutions for a low-CO_2_ cement-based materials industry. Cem. Concr. Res..

[4] Knezevic Radic J., Hansman J., Mrdja D., Demirhan K., Velimirovic D., Kuzmanovic P., Forkapic S. (2025). Survey of radiological properties of some commonly used building materials: Cement, chamotte and refractory products. J. Radioanal. Nucl. Chem..

[5] Zhang B.G. (2016). Radioactivity Measurement and Analysis of Common Building Materials in Liaoning Province. Master’s Thesis.

[6] Chen T., Jia M.Y., Chen Y.X. (2013). Determination of Radionuclide Content and Analysis of Radiation Levels in Cement. Chin. J. Radiol. Health.

[7] Tang Y.Y., Zhao F. (2020). Determination of Specific Activity of Radionuclides and Radiation Analysis in Different Types of Cement Products. Anhui Archit..

[8] Bajoga A.D., Dawaki Y.M., Seydou H. (2023). Assessment of Natural Radioactivity Levels and Radiation Hazards in Raw Materials Used for Cement Production at Ashaka Cement Company. BIMA J. Sci. Technol..

[9] Alonso M.M., Suárez-Navarro J.A., Pérez-Sanz R., Gascó C., de los Reyes A.M.M., Lanzón M., Blanco-Varela M.T., Puertas F. (2020). Data on Natural Radionuclide’s Activity Concentration of Cement-Based Materials. Data Brief.

[10] Kadum A., Bensaoula A., Dahmani B. (2013). Measurement of Natural Radioactivity in Cement of Tlemcen—Algeria by Using Well-Shape NaI(Tl) Detector. J. Nat. Sci. Res..

[11] Buranurak S., Pangza K. (2018). Assessment of Natural Radioactivity Levels and Radiation Hazards of Thai Portland Cement Brands Using Gamma Spectrometry Technique. Mater. Today Proc..

[12] (2010). Limits of Radionuclides in Building Materials.

[13] Al-Jundi J., Salah W., Bawa M.S., Afaneh F. (2006). Exposure to radiation from the natural radioactivity in Jordanian building materials. Radiat. Prot. Dosim..

[14] Beretka J., Mathew P.J. (1985). Natural Radioactivity of Australian Building Materials, Industrial Wastes and By-Products. Health Phys..

[15] European Commission (1999). Radiation Protection 112: Radiological Protection Principles Concerning the Natural Radioactivity of Building Materials.

[16] Charles M. (2001). UNSCEAR Report 2000: Sources and Effects of Ionizing Radiation. J. Radiol. Prot..

[17] (2002). Basic Standards for Protection Against Ionizing Radiation and for the Safety of Radiation Sources.

[18] (2018). Railway Concrete.

